# Charles Lynch and His Vestigial Legacy—Workplace Lynching

**DOI:** 10.5005/jp-journals-10071-23199

**Published:** 2019-07

**Authors:** Suresh Ramasubban

**Affiliations:** Department of Intensive Care Unit, Apollo Gleneagles Hospitals, Kolkata, West Bengal, India

## Abstract

**How to cite this article:** Ramasubban S. Charles Lynch and his Vestigial Legacy: Workplace Lynching. Indian J Crit Care Med 2019;23(7):289–290.

The recent attacks on doctors at NRS medical college has brought to the forefront the issue of workplace violence (WPV). The attack on the doctors in Kolkata is one more in a series of incidents involving health care workers. A type II WPV is characterised by the perpetrator being a customer of health care, however the alarm bells should be ringing all across the medical fraternity as the violence faced by the doctors in NRS, though initiated by customers, quickly transformed into a type I WPV, meaning that the perpetrators of the crime had no association with the hospital or its employees and were executing an extrajudicial order.^1^ Mob violence and lynching is a form of work place violence, which is unheard of in the developed world, and is unique to us in India.

Charles Lynch an American revolutionary, who while presiding over a Virginia court, imprisoned British loyalists without jurisdiction, lends his name to the assumption of extrajudicial authority.^2^ The persecution of African Americans after the abolition of slavery led to the peaking of lynching. However over time, lynching has undergone its natural extinction in most parts of the democratic world, except in some pockets of the world where the phenomenon continues. “Khap Panchayats” and their functioning as quasi-judicial bodies are the vestiges of this system. The dangerous vestige is of course the politically patronised group of unemployed men, who impart Lynch methodology for the gain of their masters, to provoke and shape public imagery of a particular problem and to divert attention from the real problem. In health care, the consumer, the patient or his family, assume the vestige of Charles Lynch's legacy

In this edition of the Indian Journal of Critical care Medicine, Nadikuda et al have published their study on workplace violence with a survey conducted amongst critical care physicians who're dealing with the highest level of acuity of care and the highest level of WPV. In this survey amongst young critical care doctors working in private hospital, majority of the violence was verbal, mostly at night and patient visitors were responsible for the incidents. Poor communication was perceived to be the main cause of the conflict which escalated into violence. In the private sector, billing related issues were also at the forefront. The effects on the physicians who bore the brunt of the violence was also significant with a majority of affected changing their place and pattern of work.

The survey conducted during a national conference is a voluntary retrospective survey and thus has an intrinsic bias of both selection bias and recall bias. The other issue while comparing similar surveys which have looked into perception of physicians into WPV, is the lack of a standardised instrument to measure work place violence.

Despite the limitations, the survey does for the first time highlights the perception of critical care physicians on this issue. In the western world, it's the emergency medicine doctors and nurses who bear the brunt of violence, in our countries with a nascent emergency medicine services the brunt of violence during acute emergencies are borne by the Critical care physician.

The survey also highlights the remedy that the young intensivists have for this problem. The remedies offered focus on proper communication, improving security, infrastructure and an increased responsiveness of the administration. Conflict management was an issue that every intensivist surveyed, felt as an absolute necessity. This uncannily mirrors the suggestions set forth by the junior doctors of West Bengal, when they aired their grievances on national television just a few days back. The concordance of thinking, reinforces the validity of the survey.

This issue also features another questionnaire based evaluation of factors leading to WPV, conducted in a tertiary care hospital in north India. Amongst the 295 health care workers who were screened, almost 54% reported WPV. Junior doctors faced the brunt of the violence, along with the nurses. Majority of the violence occurred during the night shift and the front line staff were the victims. The article is significant as it also addresses the view point of the perpetrators.

The potential solutions across the globe have been numerous, however none of them have any supporting evidence for decreasing WPV prospectively. The eventual solution has to be a multifaceted, multidisciplinary program, which should be customised and individualised. The strategies for mitigating and eliminating WPV has to be categorised as individual strategies, hospital strategies, law enforcement strategies and legislative strategies.

Legislative measures need to be the first cog in the wheel to decrease WPV. “ Zero tolerance policy” against violence towards heath care workers^3^ is a must and this can be achieved by the state governments passing “Hospital Protection Act” which carry significant punishments under the Indian Penal code.^4^

Law enforcement strategies by the police should focus on a quick response to distress calls from the health care workers. Violence perpetrated by an individual or a group of individuals is difficult for the police to eliminate, however a mob gathering and lynch preparation can definitely be thwarted with proper intelligence and preparedness.

Hospitals administrative strategies are the easiest to implement and may have the maximum benefit on the morale of the hospital staff. The measures include hardening of infrastructure like ensuring functioning gates/fences, security personnel, metal detectors and creating a rapid response alarm system, just to name a few. In private hospitals measures to increase staffing during busy periods, flagging potential trouble makers may be an option. For public hospitals, measures to decrease the burden on trainee doctors by hiring more post graduates senior house officers may be one way to go, however each individual hospital/medical colleges need to have their own strategy. Measures to reduce overcrowding of teaching institutions has to be addressed by all the stakeholders.

Finally, the individual, the doctor on the ground, needs to be trained in aggression de-escalation techniques, and other methods of conflict management as advocated by the authors. Communication skills, especially communication of bad news is a skill, which an individual doctor needs to master. Training in self-defence to ensure safety at workplace is also an important step that an individual needs to take.

The real problem, which if anybody cares to listen to now, is the lack of trust in the delivery of health care in this country. There is very little trust in the ethics, oaths and codes that we have set up for ourselves. This trust deficit stems from, a defunct, overburdened public health system, an unorganised, unscrupulous, corrupt private system of health care, a growing mass of aspirational, impatient citizens who are not prepared for any health related contingency, and a combination of all these factors.

This trust deficit can be eliminated only by us taking the responsibility for patient advocacy. Advocacy means to plead for the patients right to good health. The advocacy style may vary in public and private hospitals but the purpose remains the same, we should ensure the welfare of our patients. We need to take a pledge, like the fellowship pledge of the American college of Surgeons; “place the welfare and right of the patient above all else”.^5^ This is the only solution, which will be everlasting, all barriers, policing, legislation will fall flat on its face, if the trust deficit isn't wiped out.
